# Effects of Combination of Functional Electric Stimulation and Robotic Leg Movement Using Dynamic Tilt Table on Walking Characteristics in Post-Stroke Patients with Spastic Hemiplegia: A Randomized Crossover-Controlled Trial

**DOI:** 10.3390/jcm11236911

**Published:** 2022-11-23

**Authors:** Koki Ueda, Yasunori Umemoto, Yoshi-ichiro Kamijo, Yuta Sakurai, Shohei Araki, Masato Ise, Izumi Yoshioka, Motohiko Banno, Satoshi Mochida, Takaya Iwahashi, Toshio Shimokawa, Yukihide Nishimura, Fumihiro Tajima

**Affiliations:** 1 Department of Rehabilitation Medicine, School of Medicine, Wakayama Medical University, Wakayama 641-8509, Japan; 2 Department of Rehabilitation Medicine, Dokkyo Medical University Saitama Medical Center, Saitama 343-8555, Japan; 3 Nachi-Katsuura Research Center of Sports Medicine and Balneology, Nachikatsuura Balneologic Town Hospital, Wakayama 649-5331, Japan; 4 Clinical Study Support Center, Wakayama Medical University Hospital, Wakayama 641-8509, Japan; 5 Department of Rehabilitation Medicine, School of Medicine, Iwate Medical University, Morioka 028-3695, Japan

**Keywords:** walking speed, 10 m walking test, spasticity, cerebrovascular accident, head-up tilt

## Abstract

Background: Spastic hemiplegia causes slow and unstable walking in post-stroke patients. Dynamic tilt table with robotic leg movement (DTTRLM) is safe and effective in improving walking. Functional electric stimulation (FES) improves walking speed in post-stroke patients with spastic hemiplegia. The aim of this study was to determine the effects of combined DTTRLM + FES on walking speed compared with DTTRLM alone. Methods: Twenty post-stroke patients were randomly assigned to receive either a single session of stepping + FES treatment or a single session of stepping alone treatment. After a one-week washout period, the same two groups underwent a single session of the other treatment, and the same measurements were taken. We measured walking speed, cadence, and the number of steps in a 10 m walking test (10MWT) and assessed Modified Ashworth Scale (MAS), Fugl–Meyer Assessment (FMA), and range of motion (ROM) before and after the intervention. Results: Stepping + FES significantly improved walking speed, number of steps, and ankle inversion ROM, compared with stepping alone. Adverse events were not observed in any subject. Conclusions: Robotic stepping therapy combined with FES significantly improved 10 m walking speed (10MWS) compared with stepping only in patients with post-stroke and spastic hemiplegia. Further studies are needed to determine the long-term effects of the combination treatment.

## 1. Introduction

Recent advances in medical biotechnology have improved the survival rate of patients with cerebrovascular disease [1]. Activities of daily living and quality of life in patients with stroke correlate with walking speed [2,3]. The latter is negatively affected by spasticity [4], which is present in approximately 35% of post-stroke patients [5]. Spasticity causes spastic movement disorders, slowed gait, and disturbances in voluntary movement [6]. The standard treatment to improve walking ability in spastic hemiplegia after stroke is gait training [7,8,9,10,11,12]. In addition, oral medications, injection of botulinum into spastic muscles, nerve block, and surgical therapy such as tendon transfer are currently available to attenuate spasticity in the hemiplegic leg [13,14,15]. However, a large proportion of these patients are anxious about falls during walking, even those who receive one or more of the above therapies. Therefore, new rehabilitation treatments are needed to improve spasticity in the hemiplegic leg and walking ability.

The concept of robotic rehabilitation has emerged in recent years and the application of the dynamic tilt table with robotic leg movement (DTTRLM) is safe and effective in improving walking ability. The DTTRLM has been used in patients with various neurological disorders (e.g., stroke, spinal cord injury, and traumatic brain injury), trauma, cardiovascular disorders, and postoperative patients, and its beneficial effects have been confirmed [16,17,18,19,20]. Briefly, in the DTTRLM, the patient stands while strapped to the vertically tilted table and is encouraged to perform passive/active stepping, mimicking walking, under a load equivalent to that of stair climbing [21]. Standing and stepping exercises can be tailored to the patient’s condition and are an effective form of physiotherapy. We performed 10 min of standing and stepping exercises using DTTRLM on 10 patients with chronic stroke of at least 6 months onset and found a 12.8% increase in 10 m walking speed (10MWS).

On the other hand, functional electrical stimulation therapy (FES) is known to improve walking ability as well as spasticity control. The FES is designed to stimulate affected muscles through electric stimulation of the nerve supply. FES has been reported to improve walking speed in acute and chronic stroke patients with spastic hemiplegia [22,23,24].

To the best of our knowledge, there is no report on the combined use of DTTRLM and FES on walking ability in post-stroke patients with spastic hemiplegia. The aim of this study was to determine the effects of the combination of lower limb step-exercise using the DTTRLM + FES on walking in post-stroke patients with spastic hemiplegia.

## 2. Materials and Methods

### 2.1. Research Design

Interventional randomized crossover-controlled clinical trial.

### 2.2. Study Participants

The study participants were both outpatients and inpatients with clinically-confirmed stroke diagnosed at least 6 months before the study. The following were the inclusion criteria for participation: (1) minimum age of 20 years; (2) history of stroke of more than 6 months; (3) ability to walk independently with or without the use of braces; (4) greater than 1 on Modified Ashworth Scale (MAS) for the hemiplegic upper and lower extremities; (5) ability to state verbally any adverse events; (6) body weight ≤135 kg, height ≤ 210 cm, and leg length (plantar side of the foot to the greater trochanter) of 75–100 cm); (7) no bone fracture; and (8) provision of a signed consent form. We also used the following exclusion criteria: (1) history of subarachnoid hemorrhage; (2) sub-tentorial lesions on head CT or head MRI at the time of stroke; (3) inability to use the DTTRLM device and undergo FES because of skin, bone, or cartilage problems; (4) use of cardiac pacemaker, electrical stimulator, or implantable medical pump; (5) pregnancy; (6) presence of uncontrollable pain; (7) history of epilepsy; (8) bilateral hemiplegia; (9) use of stepping exercise with a robotic tilt table or FES within 1 week of enrollment; (10) botulinum toxin therapy within 4 months of enrollment; (11) uncontrolled cardiovascular or respiratory condition; (12) recent history of dizziness, cold sweating, or nausea; (13) fever of ≥38 °C; and (14) considered not suitable for participation based on clinical assessment by the attending physician.

### 2.3. Study Protocol

The study was conducted at Nachi-katsuura Balneology Hospital and Wakayama Medical University Medical Center for Health Promotion and Sport Science Satellite Clinic Honmachi in Wakayama. Randomization was achieved using computer software; participants who passed the screening inclusion and exclusion criteria were assigned at random into two groups at a 1:1 ratio. Data for outpatients were collected on the study days upon arrival of the patient to the hospital, while those of inpatients were collected in the evening of the completion of training. Patients of the first group received one session of the combination of DTTRLM-stepping + FES treatment followed by a one-week washout period, and then underwent one session of DTTRLM-stepping only treatment on the DTTRLM. Patients of the second group received one session of DTTRLM-stepping only treatment followed by a one-week washout period and then received one session of DTTRLM-stepping + FES treatment.

Each group received the study treatment assigned as the first treatment within 28 days of enrollment. Before the treatment, a 10 m walking test (10MWT) was conducted, together with measurements of MAS, range of motion (ROM) of the knee and ankle joints, and the Fugl–Meyer Assessment (FMA) of the paralyzed side. Immediately after the end of the treatment, the same parameters were measured again to determine post-intervention values. After each treatment, safety was assessed during the 7-day (+7 days) washout/observation period.

### 2.4. Intervention

The DTTRLM and FES (Erigo Pro, Hocoma AG, Volketswil, Switzerland) were used in the present study. DTTRLM is a robotic dynamic tilt table that allows functional mobilization by providing gradual verticalization combined with robotic leg movement, cyclic leg loading, and FES in patients with neurological diseases. The patient’s upper body is secured by a harness that fixes the chest and pelvis to the table. The feet are secured to mobile footplates for computer-controlled leg movement.

After immobilization on the DTTRLM, patients of the stepping + FES group were gradually verticalized from 0° to 80° (verticality to 90° results in forward slump and an unstable standing position), and stepping was initiated at a rate of 16 steps/min for 10 min. The DTTRLM device was set at 50% of the guidance force (i.e., the force required to correct deviations from the desired movement pattern. The guidance force can be set from 0 to 100%. With a low force value, the patient must increase physical effort in order to adhere to the desired movement pattern). As the DTTRLM device moved their lower extremities automatically, the participants were instructed to generate enough effort to achieve the stepping. The gastrocnemius and tibialis anterior muscles were prepared for FES using surface electrodes. FES was set to a frequency of 35 Hz, pulse rate of 280 μs, and ramp of 3 (ramp is the number of pulses to reach the target pulse width). FES-induced muscle contraction was checked visually and stimulation intensity was adjusted taking into consideration the maximum intensity of tolerance of pain for each subject.

Treatment of the stepping group was limited to standing load and lower extremity stepping exercises only (no FES).

### 2.5. Measured Variables

The primary outcome was the change in 10MWT, defined as the difference in walking speed before and after intervention in the stepping + FES group and stepping group [25]. Each participant was asked to walk a 14 m straight course at the maximum possible speed. To avoid the acceleration and deceleration phases of the test walk, the start and end times were set at the 2 and 12 m marks, respectively. The walking speed was calculated as the time elapsed while traversing the inner 10 m. The number of steps taken during the 10MWT was also counted. Measurements were taken twice. The first measurement was taken after a 10 min rest, while the second measurement was taken after 3 min of rest. The average of the two measurements was calculated. Cadence (steps per minute) was calculated from the average number of steps and the average time in the 10 m section.

Secondary outcomes were an improvement in MAS (spasticity) of the paralyzed lower extremity at the knee and ankle joints; increased cadence; increased number of steps; improvement in the lower extremity items of the FMA (34-point scale) [26]; and ROM of the knee, ankle, and foot joints.

### 2.6. Sample Size Estimates

For this study, we expected a 30% improvement in walking speed, according to a similar report by Park et al. [23]. The minimum number of cases required for power 1-β to be greater than 0.9 was 19 when evaluated at a significance level of α = 0.05. Considering a dropout rate of 5%, 20 cases were selected.

### 2.7. Statistical Analysis

The R version 4.1.3 (R Foundation for Statistical Computing, Vienna, Austria) was used for data analysis. This study used the mixed effect model to analyze the crossover trials. For treatment method (treat), order of treatment (period), group (sequence), and extent of change, we treated the observed values before each period as the fixed effects and subjects as the random effects. All data were expressed as mean ± SD. A *p*-value < 0.05 was considered to denote the presence of statistical significance.

## 3. Results

### 3.1. Participant Flowchart

**Figure 1:**
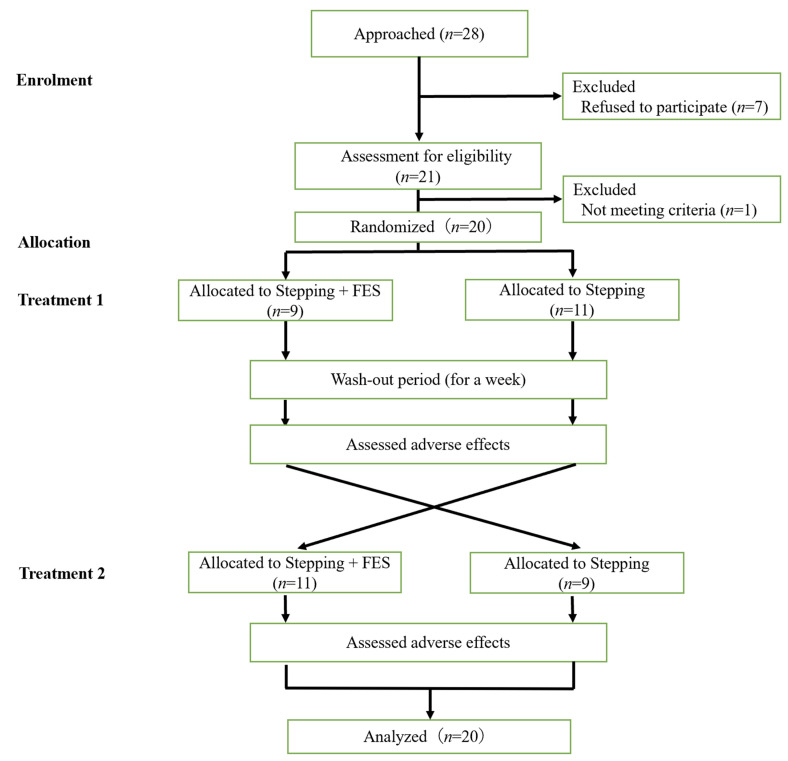
Chart of the recruitment and randomization procedures.

The study started in October 2021 and ended in March 2022. Figure 1 shows the Consolidated Standards of Reporting Trials (CONSORT) diagram. We invited 28 subjects to participate in the study; 7 refused participation, while 21 were screened and 1 was excluded. The 20 participants (11 inpatients and 9 outpatients) were randomly assigned into the two treatment groups. All participants completed all tests; thus, the data were intention-to-treat analyzed from all randomized participants. The characteristics of the participants are shown in Table 1.

### 3.2. Results of 10 m Walking Test

**Figure 2:**
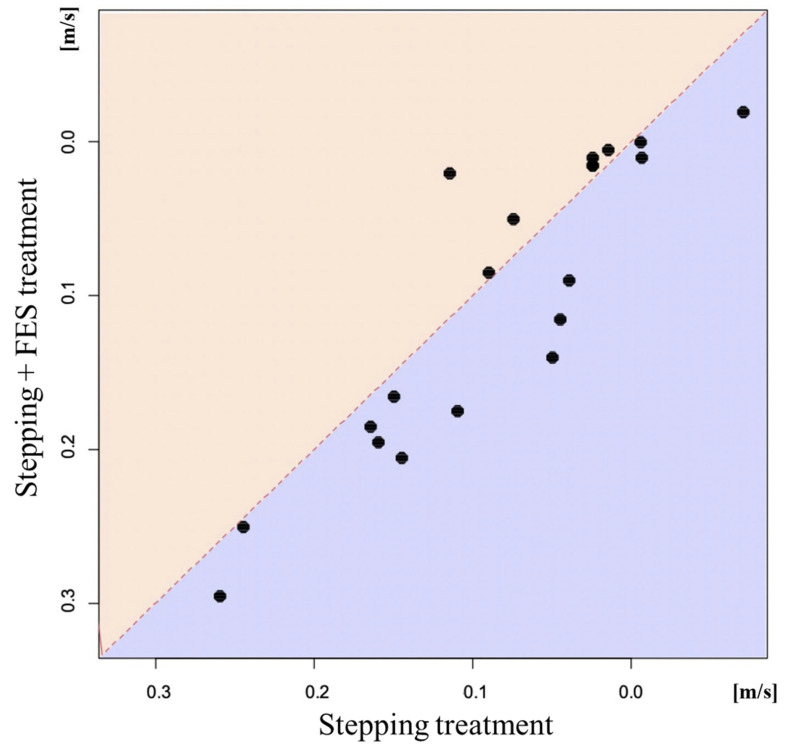
Scatter plot to compare changes in 10 m walking speed (10MWS) after stepping + FES treatment and stepping alone treatment in the same patients. The ordinate represents changes in 10MWS after stepping + FES treatment and the abscissa represents changes in 10MWS after stepping alone. Points above the line *y* = *x* denote better improvement after stepping alone, while those below the line indicate better improvement in the stepping + FES group.

The results of the 10MWT are shown in Table 2. The stepping + FES treatment induced significant changes in the 10MWT, number of steps, walking speed, and cadence. On the other hand, the stepping group showed significant changes only in the 10MWS and cadence. Figure 2 shows a scatter plot that compares walking speed after the stepping + FES treatment compared with the stepping treatment in the same participants. The stepping + FES treatment significantly improved walking speed compared with the stepping alone treatment based on the mixed effect model. Furthermore, the same statistical analysis showed a significant increase in the number of steps after the stepping + FES treatment compared with the stepping alone treatment. These improvements did not have a carry-over effect. There were no significant differences in cadence between the two groups according to the mixed effect model.

### 3.3. Changes in Range of Motion, Fugl–Meyer Assessment Score, and Modified Ashworth Scale

Table 3 shows the effects of each treatment on ROM of each joint and the FMA score. Significant improvements were noted in ROM for knee extension, ankle dorsiflexion, ankle inversion, and eversion after the stepping + FES treatment. Similar changes were observed in ROM for knee flexion, ankle dorsiflexion, ankle plantarflexion, and ankle eversion in the stepping group. Furthermore, the FMA score improved significantly by both treatment modalities; the mixed effect model showed no significant difference in the FMA between the two groups. However, the same analysis showed a significant difference in ROM for ankle inversion between the two groups. Both stepping + FES and stepping alone resulted in significant improvements in MAS of knee flexion and ankle dorsiflexion.

### 3.4. Current Intensity

The current intensity of the FES used for the gastrocnemius muscle was 31.9 ± 6.3 mA, while that used for the tibialis anterior muscle was 23.5 ± 5.7 mA. None of the subjects reported adverse events in both arms of the study.

## 4. Discussion

The main finding of the present study was the significant increase in 10MWS following treatment of post-stroke patients with the combination of robotic therapy/stepping + FES compared with stepping alone. This finding suggests that the combination of FES and stepping exercise can have immediate beneficial effect on gait speed in post-stroke patients with spastic hemiplegia. Other findings of our study included the following: (1) significant decrease in the number of steps in the stepping + FES group compared with the stepping alone group, (2) improvement in 10MWS after both treatment modalities, (3) significant increase in MAS of ankle dorsiflexion in both treatment protocols, and (4) significant expansion in ROM of inversion in the ankle joint following stepping + FES compared with stepping alone. Our results demonstrated for the first time the beneficial effects of the combination of stepping + FES on 10MWS compared with the stepping alone.

Previous studies reported an improvement in walking speed in post-stroke patients after alternating lower limb exercises, such as cycle ergometer exercises and single-step training [27,28,29]. The significant increase in walking speed observed in the present study following both treatment protocols was in agreement with the above studies. The reason for the improvement in walking speed in both groups might be related to the observed improvement in dorsiflexion MAS. Spasticity is one of the major factors known to contribute to the decrease in walking speed and active exercise is known to decrease MAS [30,31] and improve walking speed in patients with cerebral palsy [31].

What is the mechanism(s) of the observed increase in walking speed after the stepping + FES treatment? While we did not examine this issue in detail, one possible explanation for the beneficial effects of the combination of DTTRLM and FES is the expansion of ROM of inversion of the ankle joint. It has been reported that FES of both the paretic ankle plantar flexors as well as the dorsiflexors provided the advantage of greater swing-phase knee flexion, greater ankle plantarflexion angle at toe-off, and greater forward propulsion, compared with FES of only the ankle dorsiflexor muscles during the swing phase [32]. The present finding adds support to those of the above study. Stimulation of the gastrocnemius muscle as well as the tibialis anterior muscle may lead to an increased walking speed through the effects of stepping out of the ankle joint by improving the ROM of inversion of the ankle joint. Stepping using DTTRLM significantly increased ROM, while FES seems to have an additional effect on the improvement in ROM. It is possible that FES might have other physiological effects, such as inhibition of spinal and/or higher cortical or other neurons.

Another possible mechanism of the observed increase in walking speed in the stepping + FES group could be an increase in motor cortical excitability. Previous studies demonstrated a substantial decrease in corticospinal tract activity during walking in post-stroke patients, with a resultant decrease in walking speed [33]. Electrical stimulation is also reported to activate the contralateral primary sensorimotor cortex [34,35,36,37]. Furthermore, the combination of electrical stimulation and voluntary contraction is known to further increase motor cortical excitability, compared with electrical stimulation alone [38]. In the present study, we believe that FES stimulation of the paralyzed side increased the excitability of the corticospinal tract, resulting in a significant improvement in walking speed in the stepping + FES group.

Another possible mechanism of the improved walking speed in the stepping + FES group is an increased stride length. The narrow stride length of steps in patients with hemiplegia is the main factor responsible for the decrease in walking speed. The stride length increased in our patients following the DTTRLM robotic exercise combined with FES, compared with the stepping group. The increase in stride length could improve gait speed and the combination of DTTRLM robotic stepping and FES may further extend the distance the length of steps achieved during stepping.

There are several limitations in this study. The present research design was not a blind study. Owing to the nature of the intervention, neither the assessors nor participants were blinded to group allocation and intervention. The study also did not include another control group of FES only to allow the assessment of the sole effect of FES on the primary and secondary outcomes. There was also a lack of diversity among the participants.

## 5. Conclusions

We have demonstrated in the present study that the combination of assisted stepping with robotic therapy + FES applied in a single session significantly improved 10MWS in post-stroke patients with spastic hemiplegia, compared with robotic therapy alone. Further studies are needed to determine the effects of a longer duration of this treatment modality and the long-term effects of the same on gait, walking, and spastic hemiplegia.

## Figures and Tables

**Table 1:** Characteristics of subjects.

Factor	Overall (*n* = 20)	A (*n* = 9)	B (*n* = 11)
Age (years)	73 [31, 89]	72 [31, 84]	74 [55, 89]
Sex: Male, Female	8, 12	4, 5	4, 7
Body height (m)	1.53 [1.39, 1.74]	1.57 [1.41, 1.74]	1.52 [1.39, 1.73]
Body weight (kg)	51.85 [43, 80]	51.80 [44.5, 80]	53.80 [43, 77.7]
Affected side: Right, Left	10, 10	4, 5	6, 5
Lesion: Hemorrhagic, Ischemic	12, 8	6, 3	6, 5
Use of orthotics (%)	8 (40.0)	4 (44.4)	4 (36.4)
mRS score	2.00 [1, 4]	2.00 [1, 3]	2.00 [1, 4]
MAS score on affected side (0/1/1+/2/3/4)			
Knee extension	17/2/1/0/0/0	6/2/1/0/0/0	11/0/0/0/0/0
Knee flexion	10/3/7/0/0/0	4/1/4/0/0/0	6/2/3/0/0/0
Ankle dorsiflexion	0/1/10/5/4/0	0/1/2/3/3/0	0/0/8/2/1/0
Ankle plantar flexion	19/0/0/1/0/0	8/0/0/1/0/0	11/0/0/0/0/0

Data are median values [minimum, maximum] or *n* (%); mRS: Modified Rankin Scale, MAS: Modified Ashworth Scale.

**Table 2:** Changes in 10MWT after the intervention.

	Stepping + FES Group		Stepping Group		Mixed Effect Model	
	Mean [95%CIs]	*p*-Value	Mean [95%CIs]	*p*-Value	Coeff [95%CIs]	*p*-Value
10 m walking time (s)	−1.35 [−0.59, −2.12]	0.001	−0.59 [0.62, −1.80]	0.318	−0.68 [0.57, −1.88]	0.306
10 m walking steps (step)	−1.02 [0.00, −2.05]	0.05	0.17 [1.48, −1.13]	0.783	−1.18 [−0.09, −2.18]	0.026
10 m walking speed (m/s)	0.10 [0.14, 0.06]	<0.001	0.08 [0.12, 0.04]	<0.001	−0.02 [−0.04, 0.00]	0.048
Cadence (step/min)	6.69 [3.05, 10.32]	0.001	7.84 [4.72, 10.97]	<0.001	−1.38 [2.35, −5.08]	0.461

Data are mean values [95% confidence interval] and coefficient of determination [95% confidence interval]; FES: functional electrical stimulation.

**Table 3:** ROM and FMA scores before and after the intervention.

	Stepping + FES Group			Stepping Group			Mixed Effect Model	
	Before	After	*p* Value	Before	After	*p* Value	95%Coefficient Interval	*p* Value
ROM (degree)								
Knee extension	2.75 ± 3.02	3.75 ± 3.19	0.042	2.00 ± 4.10	3.00 ± 4.10	0.214	0.16 [1.51, −1.15]	0.817
Knee flexion	148.5 ± 9.1	148.8 ± 9.2	0.666	147.5 ± 8.4	149.2 ± 7.8	0.015	−1.50 [0.13, −3.38]	0.089
Ankle dorsiflexion (knee extension position)	4.25 ± 7.12	10.25 ± 8.03	<0.001	4.75 ± 5.50	10.25 ± 5.95	<0.001	0.34 [2.39, −1.86]	0.748
Ankle dorsiflexion (knee flexion position)	17.25 ± 9.52	24.25 ± 8.63	<0.001	16.00 ± 7.88	22.00 ± 8.01	<0.001	1.22 [3.75, −1.25]	0.343
Ankle plantar flexion	58.75 ± 11.68	61.75 ± 7.48	0.062	60.25 ± 7.16	63.00 ± 7.68	0.001	−0.18 [2.50, −2.71]	0.892
Ankle inversion	48.25 ± 11.84	53.50 ± 8.44	0.007	45.25 ± 10.94	45.75 ± 9.22	0.776	6.39 [9.86, 2.69]	<0.001
Ankle eversion	4.75 ± 10.19	9.00 ± 8.37	0.009	4.50 ± 6.26	8.25 ± 5.20	0.001	0.74 [3.59, −1.92]	0.612
FMA Score	23.90 ± 4.68	25.65 ± 4.74	0.001	24.20 ± 5.34	26.10 ± 4.42	0.001	−0.11 [0.94, −1.17]	0.834

Data are mean ± SD. FMA: Fugl–Meyer assessment.

## Data Availability

The datasets generated during the current study are available from the corresponding author upon reasonable request.

## Abbreviations

DTTRLM: dynamic tilt table with robotic leg movement.
